# Design of Biomass Adsorbents Based on Bacterial Cellulose and *E. crassipes* for the Removal of Cr (VI)

**DOI:** 10.3390/polym17121712

**Published:** 2025-06-19

**Authors:** Uriel Fernando Carreño Sayago, Vladimir Ballesteros Ballesteros, Angelica María Lozano

**Affiliations:** Fundación Universitaria los Libertadores, Bogotá 111221, Colombia; vladimir.ballesteros@libertadores.edu.co (V.B.B.); amlozanoa@libertadores.edu.co (A.M.L.)

**Keywords:** *Crassipes*, cellulose bacterial, isotherms, adsorbents, Cr (VI)

## Abstract

Cellulose has been identified as a medium for heavy metal removal due to its high adsorption capacity in relation to these contaminants. Furthermore, cellulose is abundant and can be obtained in a practical and easy way. A notable example is *E. crassipes* biomass, which is abundant in wetlands and has not yet been efficiently and sustainably removed. Another biomass that has been used in heavy metal removal projects is bacterial cellulose. Generating this biomass in a laboratory setting is imperative, given its 100% cellulose composition, which ensures optimal adsorption capacities during the development of heavy metal adsorbent systems. Therefore, the objective of this project was to design biomass adsorbents that combine the properties of bacterial and *E. crassipes* cellulose for Cr(VI) removal. The rationale for combining these two materials is based on the premise that it will produce optimal results, a hypothesis supported by the documented efficiency of bacterial cellulose and the formidable resilience of *E. crassipes* biomass to elution processes. The second-order model and the Langmuir isotherm fit proved to be the most suitable, indicating that there an occurred interaction between the adsorption sites of these biomasses and Cr (VI). This suggests the presence of a significant number of active sites on the surface of these materials. The EC(50)+BC(50) biomass, with an adsorption capacity of 42 g of Cr(VI) per dollar, is the most cost-effective due to the low cost of *E. crassipes* and the high capacity of bacterial cellulose. It is a mixture that guarantees high adsorption capacities and facilitates up to seven reuse cycles through elutions with ethylenediaminetetraacetic acid (EDTA). This finding emphasizes the potential of this material for implementation in environmental sustainability initiatives, particularly those focused on the removal of heavy metals, including Cr(VI).

## 1. Introduction

In the contemporary era, there is growing demand for research that can provide novel and innovative solutions to the pressing challenges posed by water resource management. This need arises as a result of the observed decline in ecosystem services globally, a phenomenon that can be attributed to the dissemination of heavy metals into water systems through various industrial activities [1,2,3,4]. The presence of heavy metals such as chromium (VI) in water bodies has the potential to pose a significant threat to human health, particularly in communities that have direct contact with these materials, such as those employed in tannery companies [5,6,7]. Chromium (VI) toxicity has been shown to induce genetic alterations in human cells, underscoring the critical need for effective risk mitigation strategies [8,9]. To address these challenges, a major research initiative is focusing on the use of adsorbent biomasses, which have proven to be highly effective in removing these metals from water sources. Their ease of synthesis and economic viability make them a particularly attractive solution [10,11]. Removal, reuse, and elution processes give rise to complex mathematical models that facilitate a deeper understanding of the underlying mechanisms. These models also serve to improve the scalability of treatment processes, crucial in addressing the global water crisis [12,13]. The most widely used models are isotherms, amongst which the Langmuir and Freundlich isotherms are the most widely used [14,15,16]. Adsorption kinetic models help understand the dynamics of coupled adsorption and determine the trajectory of the process [17,18,19]. Modelling equilibrium adsorption data using isotherm equations is the most convenient and widely used method for investigating adsorption mechanisms, maximum adsorption capacities, and the properties of adsorbent biomasses, where they serve as critical design models in the construction of scaled systems [20,21,22,23,24]. Kinetic models are also presented. These help to determine the adsorption rate constants, which are decisive in scale-up processes. The kinetic study of adsorption, i.e., equilibrium adsorption, provides information on the adsorption rate, the performance of the adsorbent used, and the mass transfer pathways [16]. *E. crassipes* is a biomass that is widely used in adsorption processes of heavy metals, dyes, and phenols in research related to industrial effluent pollutants due to two key factors: its abundance and its easy availability. Its biomass contains around 30% cellulose, 33% hemicellulose, 20% lignin, and 10% of other components [25,26]. Lignin contributes to its stability and helps it to withstand the elutions of ethylenediaminetetraacetic acid (EDTA). This enables reuse more than seven times, which increases its viability in the removal of heavy metals [27]. *E. crassipes* biomass exhibits a complex reticular structure, being composed of a matrix that can interact with heavy metals [28]. The other biomass used in this wastewater process is bacterial cellulose, which is 100% environmentally friendly thanks to its sustainable and simple production process [29]. Bacterial cellulose is a polymer obtained through fermentation with microorganisms of the genera *Acetobacter*, *Rhizobium*, *Agrobacterium*, and *Sarcina.* Among these, *Acetobacter xylinum* has been identified as the most efficient species [30]. This biomass exhibits a chemical structure analogous to that of plant-derived cellulose, but differs in its conformation and physicochemical properties [31]. Notably, this cellulose lacks lignin, a characteristic that makes it particularly suitable for heavy metal adsorption processes, attributable to its greater number of active sites [32]. The cellulose content of this biomass ensures active sites for cation exchange between H^+^ and heavy metals present in the water, resulting in high heavy metal retention capacities: 69 mg/g for Cd(II) and 100 mg/g for Cr(VI) [32]. Notably, bacterial cellulose biomass exhibits industrial potential, although it is more difficult to obtain, with a 10-fold higher yield than plant cellulose biomass [33]. Consequently, there are still gaps in the research on bioadsorbents. These must be addressed by finding parameters that overcome the drawbacks in the implementation of biomasses to remove heavy metals. The combination of *E. crassipes* biomass (EC) and bacterial cellulose (BC) is proposed to determine the potential to create an ideal composite material, given the high capacity of BC biomass combined with the resilience of EC biomass. The present project was initiated with the objective of designing biomass adsorbents between bacterial cellulose and *E. crassipes* cellulose for the removal of Cr(VI) present in industrial wastewater.

## 2. Materials and Methods

We utilized *Eichhornia crassipes*. The taxonomic classification of this species is *Eichhornia crassipes*. The collection site is situated within the municipality of Mosquera, situated on the outskirts of Bogotá D.C., Colombia, with precise geographical coordinates of 4.682995, −74.256673. The collection comprised 60 dead water hyacinth plants. The *E. crassipes* plants were meticulously washed, with a particular focus on the roots and leaves. The plants were sun-dried for three days, after which they were ground to a maximum diameter of 0.212 mm using a knife mill and sieved through a 70-mesh sieve (US standard sieve). A yield of 97% was obtained, with the remaining 3% was discarded as leftover material.

Bacterial Cellulose Production: The bacterial cellulose production process is described below. Bacterial cellulose (BC) films were produced using a tea and sugar culture medium obtained from the bioprocess laboratory of Universidad Fundación Los Libertadores, Bogotá D.C., Colombia, Bacterial cellulose (BC) production involved the use of red tea, common sugar (3 g), and commercial yeast (composed mainly of the species *Saccharomyces cerevisiae*) (5 g), which were dissolved in distilled water (1 L). The mixture was subsequently heated to 80 °C to form the initial culture medium. Then, approximately 420 mL of kombucha sediment (composed of a symbiotic culture of bacteria and yeast) was added to a 3 L glass container and placed in a 15 L incubator (IO Xtemp Series Dual Incubator Oven, Hettich, Föhrenstr, Germany) at a temperature of 37 °C. pH, humidity, and temperature constant across samples. Cellulose films are produced within approximately two weeks. Subsequently, the BC films are subjected to a drying process in order to remove excess water. Following this, the cells are washed in distilled water containing 0.5 M sodium hydroxide (NaOH), with the purpose of removing excess cells and fermentation residues. After drying the bacterial cellulose films, they were ground to a maximum diameter of 0.212 mm using a knife mill and sieved through a 70-mesh sieve (US standard sieve). A yield of 96% was obtained, the remaining 4% was discarded as leftover material.

Batch adsorption experiments. The 1000 (mg/L) Cr(VI) stock solution was prepared with distilled water using potassium dichromate (K_2_Cr_2_O_7_). This stock solution was used to prepare the 200; 300; 400; and 600 mg/L Cr(VI) test solutions. The batch adsorption study was carried out at a temperature of 24 °C. The adsorption capacity was determined by suspending 0.3 g of biomass in 100 mL of Cr(VI) solution for 550 min at 200 rpm. Experiments were carried out in a 100 mL glass vessel with constant stirring (IKA KS 4000 stirrer, Staufen, Germany). Data were recorded at 20 min intervals until 180 min had elapsed. All experiments were performed in triplicate and the mean values were calculated for the final analysis. In this study, the tests were conducted under neutral pH conditions, favours the adsorption process in this type of biomass.

Chromium Determination: Samples were taken at each time interval and analyzed to assess the residual chromium concentration. Twenty-microliter samples were obtained and subsequently centrifuged (KASAI MIKRO 200). Diphenylcarbazide solution is composed of diphenylcarbazide (1,5-diphenylcarbazide) and a solvent. Acetone is commonly used as the solvent. Then, 200 microliters of 0.5% diphenylcarbazide (97% purity) solution was prepared using 120 microliters of acetone (97% purity, *w*/*v*) and 80 microlitres of diphenylcarbazide. Next, 900 microliters of phosphate buffer was prepared, prefiltered, adjusted to a pH of 2 and a purity of 90% (H_3_PO_4_). Subsequently, 100 microliters of the residual sample was added to an Eppendorf tube. An appropriate portion was transferred to an absorption cell, where the absorbance was measured at 540 nm. The measurement uncertainty of the study indicates that measurements of heavy elements, specifically Cr(VI), can be performed with an uncertainty level of approximately 4%.

Chromium Measurement: Chromium content was measured with a spectrophotometer (Evolution 300 spectrophotometer) by monitoring changes in light absorption. All procedures for determining chromium in water and substrates were carried out following the APHA (American Public Health Association) procedures for standard analysis (Standard Methods for the Analysis of Water and Wastewater).

### 2.1. Creations of Biomass Adsorbents

100% *E. crassipes* (0.3 g) (*w*/*w*): EC

75% *E. crassipes* (0.225 g) and 25% cellulose bacterial (0.075 g) (*w*/*w*): EC(75)+BC(25)

50% *E. crassipes* (0.150 g) and 50% cellulose bacterial (0.150 g) (*w*/*w*): EC(50)+BC(50)

25% *E. crassipes* (0.075 g) and 75% cellulose bacterial (0.225 g) (*w*/*w*): EC(25)+BC(75)

100% Cellulose bacterial (0.3 g) (*w*/*w*): BC

Desorption–Adsorption: The Cr(VI) adsorption process was followed by an elution process. The eluent solution was prepared with 0.3 g of EDTA in 100 mL of distilled water in a 200 mL Erlenmeyer flask; the Cr(VI)-loaded biomass was placed in this container at 25 °C for 2 h with constant stirring. Subsequently, the biomass was separated by a filter [33]. Subsequently, these biomasses, treated with EDTA, underwent other adsorption processes using 600 mg/L Cr(VI).

ATR-FTIR: The materials were characterized by Fourier Transform infrared spectroscopy (79 Jasco FTIR 430) (Tokyo, Japan) to measure IR spectra in a spectral range of 4000–400 cm^−1^ at a resolution of 4 cm^−1^ and a scanning speed of 2 mm s^−1^. They were also assessed by electron microscopy before and after the adsorption process.

SEM and EDS Analysis: The results observed in investigations involving cellulose and heavy metals were confirmed by SEM and EDS analysis using the TESCAN FE-MEB LYRA3 Focused Ion Beam Scanning Electron Microscope (Brno, Czech Republic). The device features an integrated microanalysis system for Energy-Dispersive X-ray Spectroscopy (EDS).

### 2.2. Model Evaluation

Isotherm models are utilized to predict the adsorption capacity of heavy metals in adsorbent biomasses. The most representative models are the Langmuir and Freundlich models [20], and these are complemented by adsorption kinetics. Through these, the conditions in which the adsorption model is represented can be established, thereby enabling the optimal operating conditions of adsorption to be determined. The pseudo-second-order model is considered a special type of Langmuir kinetic model because it takes into account how the reaction rate depends on the amount of metal ions on the surface of the biomass and the amount of adsorbed metal when this biomass is saturated [16]. In the Table 1 shows the Models of isotherm and kinetics.

### 2.3. Measurement of the Pore Volume of Biomass

The density of the adsorbent biomass is calculated using the elementary density equation.

(6) $$\rho =\frac{m}{V}$$

where (V) is the volume (mL); this includes spaces between pores and air and is a fundamental parameter. Total biomass (m) occupied in the adsorbent site is determined, where m is the amount of biomass (g) used in the process of the adsorption of heavy metals.

(7) $$\rho (p)=\frac{m(p)}{V(p)}$$

The particle density ρ(p) is obtained, which is used directly in the metal adsorption process. The mass m(p) of the particle occupying a space V(p) includes all spaces; this volume can be calculated using Equation (8).

(8) $$Vp=\frac{4\pi r^{3}}{3}$$

where V(p) is obtained from the diameter of the small particle (r), assuming that it is spherical. In experiments with adsorbent biomass, it has been established that the diameter should not exceed 0.212 mm [34].

(9) $$\varepsilon =1-\frac{\rho }{\rho (p)}$$

Parameter (ε) relates these two densities, the density of the biomass and the density of the particle. Its best performance is 0.5–0.8 [35].

## 3. Results

### 3.1. Measurement of Biomass Pore Volume

To achieve the desired performance, it is essential to maintain the particle diameter at 0.212 mm or less. Using this value, the volume of each particle was calculated using Equation (8) [34,35]. In the Table 2 shows the results of the analysis of the relationship between densities.

Equations (6)–(9) were used to determine the characteristic parameter of the density ratios (ε) for each biomass used in the adsorption processes. The mass (m) was constant for all biomasses; however, the volume (V) varied depending on the biomass. For example, bacterial cellulose occupied 0.48 mL, while *E. crassipes* biomass occupied a smaller volume of 0.35 mL. Consequently, the densities of each biomass varied. The particle volume did not exceed 0.005 mm in all biomasses, determined Equation (8), while the weight of each microparticle differed depending on the specific biomass. With these two parameters, the particle density was obtained. To achieve optimal values of (ε), it is essential that the particle density be slightly higher than the biomass density, in a range of 60% to 90%. This indicates that the values of this design parameter should be between 0.4 and 0.75 [35]. A biomass with an (ε) value less than 0.4 indicates the absence of a direct relationship between the microparticle and the contaminant; on the contrary, a biomass with an (ε) value greater than 0.85 means that the biomass is compact in terms of density, resulting in insufficient space for the heavy metal adsorption process. The BC biomass demonstrated a coefficient of 0.68, which signifies an optimal relationship between its microparticle and biomass density [36,37,38,39]. A comparable result was observed for the EC(50)+BC(50) and EC(25)+BC(75) biomasses, which remained within the designated (ε) ranges. EC biomass contains lignin [40,41,42], contributing to its compact structure. This improves the target contact characteristic between the contaminant and the biomass, resulting in an increase to 0.57. A similar trend was observed in EC(75)+BC(25) biomass. The value of (ε) has been used in various investigations for the characterization of adsorbent materials. For example, Ref. [43] characterized *E. crassipes* biomass, obtaining values of 0.61. The value for cocoa (ε) is 0.68 [44], with values of 0.86 for sugarcane bagasse and 0.71 for rice residue [45]. The value for zeolite adsorbent material is 0.38 [46], with one of 0.6 for activated carbon [47].

### 3.2. Material Characterization

**Figure 1:**
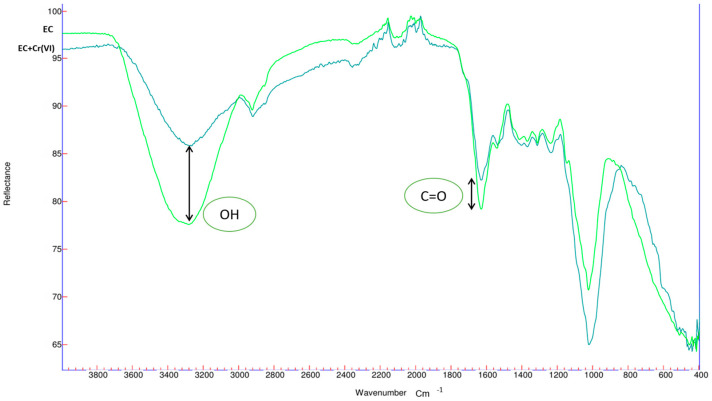
FTIR characterizations of EC.

Figure 1 presents the FTIR spectra of the biomass before and after the Cr(VI) adsorption process.

The hydroxyl (OH) groups, characteristic of this biomass and present in the 3280 cm⁻^1^ band, appear elongated due to the presence of cellulose and lignin. The organic C=O group, present in the 1651 cm⁻^1^ band, is found in proteins, amino acids, and peptides, and is frequently part of carbohydrates, lipids, and other structures [48]. Regarding the FT-IR spectra of Cr(VI)-loaded ECs, after Cr(VI) adsorption, the adsorption peaks at 3380 cm^−1^, corresponding to hydroxyls, were attenuated because these groups influenced the adsorption process [49].

**Figure 2:**
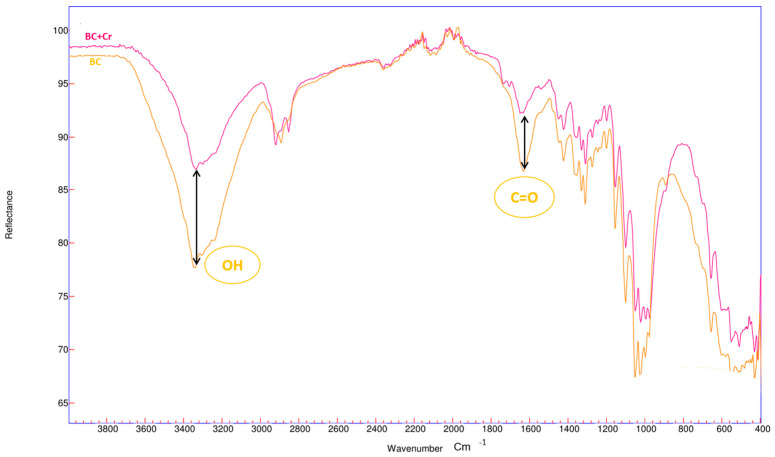
FTIR characterization of BC.

The FT-IR spectra of the original BC samples and the Cr(VI)-loaded BC were studied to examine the interaction of this heavy metal with cellulose, with results shown in Figure 2.

After the Cr(VI) adsorption experiment on BC biomass, as with EC biomass, significant changes were observed in the hydroxyl (OH) group’s peak. Furthermore, a slight shift in the peak at 1632 cm⁻^1^, corresponding to the carbonyl group C=O, was observed due to the adsorption of Cr(VI) ions; this organic group is characteristic of cellulolytic biomasses [50,51].

**Figure 3:**
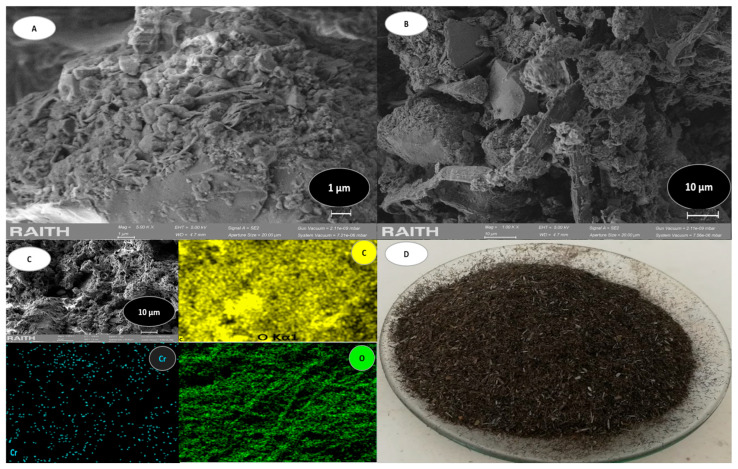
EC analysis. (**A**) shows SEM morphology at 1 µm. (**B**) shows SEM morphology at 10 µm. (**C**) shows EDS mapping that revealed distribution of elements. (**D**) shows particles of adsorbent obtained from *E. crassipes* plants.

Figure 3 shows some characteristic images of the biomasses used in the present investigation, featuring representations of the vegetal biomass of *E. crassipes* obtained using SEM and EDS analysis. This porosity is due to the quantity of lignin and hemicellulose present in this biomass [52,53,54], where the porosity of the vegetal material analyzed is evident.

The EC biomass behaves as a complex network, and a matrix is observed within it that possibly interacts with the heavy metal (Figure 3A,B). Furthermore, EDS mapping revealed the distribution of the elements carbon (C), oxygen (O), and chromium (Cr) throughout the three-dimensional structure (Figure 3C). A sample of this biomass after was obtained initial adsorption with a maximum chromium concentration of 600 mg/L. Figure 3D is an image of particles of the adsorbent obtained based on *E. crassipes* plants. EDS analysis of EC is presented in Table 3, which shows the characteristic elements in a sample of this biomass after Cr(VI) adsorption.

**Figure 4:**
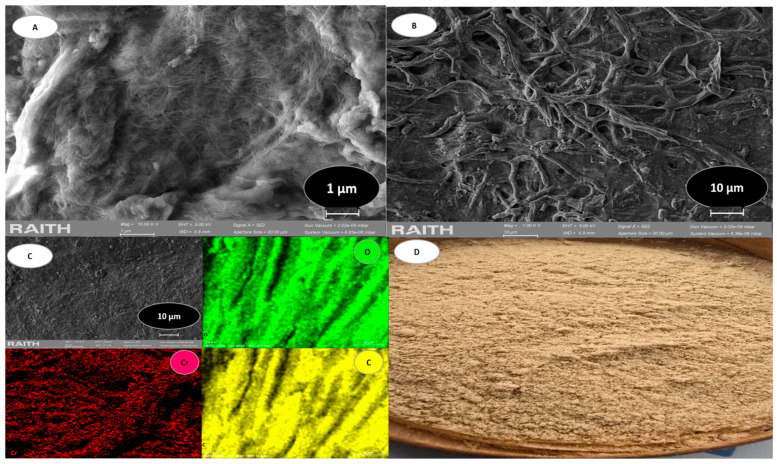
BC analysis. (**A**) shows SEM morphology at 1 µm. (**B**) shows SEM morphology at 10 µm. (**C**) shows EDS mapping that revealed distribution of elements. (**D**) is image of particles of adsorbent obtained from BC.

The EDS evaluation revealed that the aquatic plant *E. crassipes* contains substantial amounts of elements such as carbon and oxygen. The presence of other elements was detected in trace amounts. Similar results were reported in the study by [40], which also highlighted the robustness and comparable characteristics of the examined samples. The analysis of *E. crassipes* biomass is complex due to the distribution of lignin, hemicellulose, and cellulose in this plant. Since the sample was taken after the Cr(VI) adsorption process, this heavy metal accounted for 4.1% of the sample. Figure 4 shows the analysis of the BC.

This type of biomass presents rebranches, a characteristic of bacterial cellulose [55]. It is noteworthy that bacterial cellulose presents greater fibrillation than *E. crassipes* biomass. The analysis revealed the presence of nanofibers that form a three-dimensional mesh, similar to interconnected sheets. This is coupled to porous channels, which intertwine with each other in a network [56,57] (Figure 4A,B). Furthermore, after the adsorption process, a sample of this biomass was obtained after an initial adsorption with a maximum chromium concentration of 600 mg/L. EDS mapping was performed, which revealed a distribution of the elements carbon (C), oxygen (O), and chromium (Cr) along the three-dimensional structure (Figure 4C); Figure 4D is a particle image of the obtained BC-based adsorbent. The EDS analysis of BC is presented in Table 4, which shows the characteristic elements in a sample of this biomass after Cr(VI) adsorption.

As illustrated in Table 4, the biomass shows a substantial amount of Cr(VI) at 9.1%, occurring due to the chemisorption process. This is characteristic of bacterial cellulose samples.

### 3.3. Results of Remotion’s of Cr (VI)

**Figure 5:**
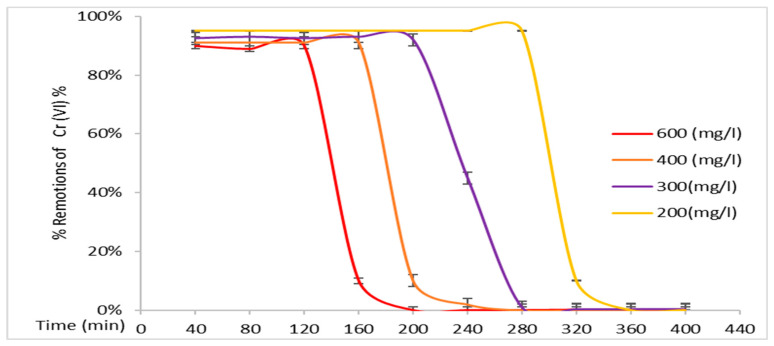
Removal percentages of Cr (VI) from EC(75)+BC(25) biomass.

The results of the adsorption process were performed in triplicate, with the plots representing the average of the three data points, and the error bars representing the standard errors. Figure 5 illustrates the removal percentages of EC(75)+BC(25) biomass.

**Figure 6:**
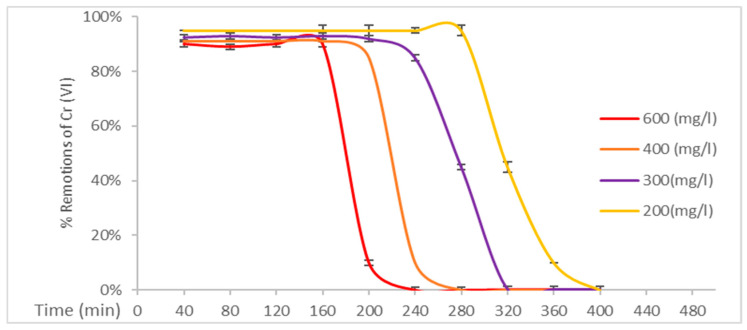
Removal percentages of Cr (VI) by EC(50)+BC(50).

It is evident that this treatment system comprises a higher percentage of vegetal cellulose biomass than bacterial cellulose. Consequently, significant removals occurred; however, they were the lowest of the experiments due to the limited amount of bacterial cellulose biomass. Noteworthy removals were observed at all initial concentrations, with 90% of Cr (VI) being removed at its maximum concentration of 600 mg/L and a treatment duration of approximately 120 min seen with this heavy metal. *E. crassipes* obtained removals of 90% at Cr (VI) concentrations of 500 mg/L [25] and removals higher than 99% from bacterial cellulose alone, performing for more than 220 min with concentrations of 500 mg/L [30]. Figure 6 demonstrates the treatment system using biomass EC(50)+BC(50).

**Figure 7:**
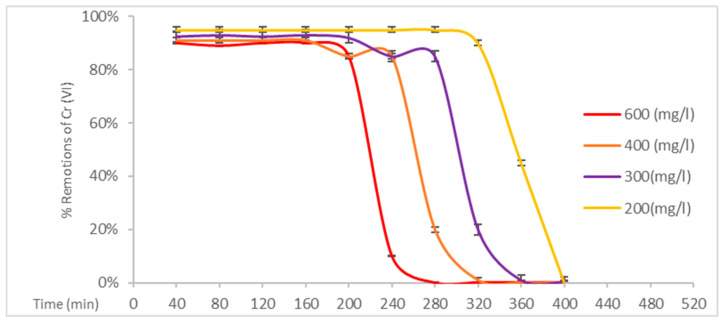
Removal percentages of Cr (VI) by EC(25)+BC(75).

The system using EC(50)+BC(50) is characterized by balanced biomass compositions. This system demonstrated optimal performance, particularly when the BC biomass was increased relative to the EC(75)+BC(25) composite material. The enhancement in the presence of cellulose resulted in augmented retention of this heavy metal, achieving a treatment duration of 160 min in water contaminated with Cr (VI) at an initial concentration of 600 mg/L. The combination of these two celluloses is optimal due to their ideal design characteristics, with BC providing more active sites for the removal of this heavy metal, and EC providing an ideal support material for future elutions [27]. Figure 7 shows the biomass treatment system EC(25)+BC(75).

As illustrated in Figure 7, with a treatment duration exceeding 200 min and initial concentrations of 600 mg/L, this composite material exhibits the optimal performance in comparison to the other two materials. Bacterial cellulose possesses an optimal cellulose content, while this polysaccharide possesses a higher number of active sites within its cellulose matrix.

### 3.4. Adsorption Capacities

The adsorption capacities were obtained through Equation (6). This equation is ideal for establishing this design parameter because it uses all the design variables of a continuous-flow biomass treatment system. The initial concentration of 600 mg/L was used, since it was the maximum concentration used. Table 5 provides a summary of the characteristic parameters of the samples analyzed in the preceding figures.

The adsorption capacity of EC biomass is 44 mg/g, whereas similar experiments have shown that BC biomass is 150 mg/g [17]. The combination of these materials is ideal because it can provide relevant information when constructing a large-scale treatment system with these biomasses [28]. For instance, when confronted with the necessity of treating contaminated water with a substantial volume and elevated contaminant levels, the EC(25)+BC(75) treatment system emerges as a viable solution, underpinned by its substantial adsorption capacity of 123 mg/g. Conversely, when the objective is to decontaminate a treatment system with a lower contaminant load but maintain the same flow rate, the installation of an EC(50)+BC(50) treatment system is recommended. In scenarios where a more economical system is desired that is less effective but achieves comparable removal rates, the implementation of an EC(75)+BC(25) treatment system is a viable option.

### 3.5. Isotherms

Adsorption isotherms represent the mathematical behaviour of the adsorption process, whereby the adsorption capacities of each concentration, in conjunction with the equilibrium concentration, furnish information regarding the model to be employed in the performance of adsorbent biomasses [9]. Isotherm evaluations of adsorption processes are of considerable utility in accurately investigating the nature and adsorption mechanisms of heavy metals in the various biomasses to be evaluated. Table 6 shows the isotherms.

**Figure 8:**
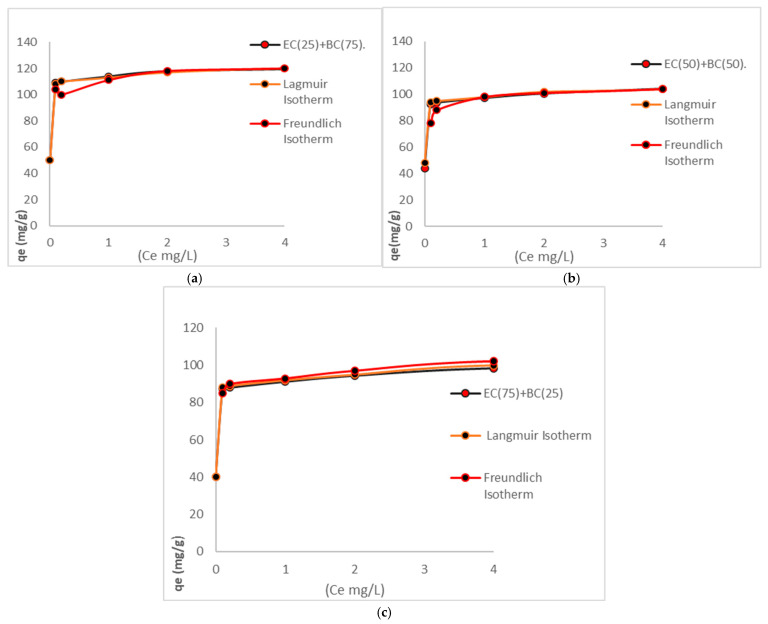
The various isotherms are represented by distinct graphs. (**a**) Represents EC(25)+BC(75) isotherm. (**b**) Represents EC(50)+BC(50) isotherm. (**c**) Represented EC(75)+BC(25) isotherm.

As demonstrated in Figure 8, the various isotherms are represented by distinct graphs, thereby facilitating the visual assessment of the adsorption capacities of each biomass. Notably, the biomass EC(25)+BC(75) exhibits superior performance in terms of adsorption capacity, attributable to its increased content of bacterial cellulose. The graphs in Figure 8 offer a quantitative comparison between the adsorption capacities (qe) and the equilibrium concentrations (C_e_). It is important to note that each isotherm is characterized by its own unique graph, as delineated by the representative model. Through the utilization of equilibrium results, a comparative analysis was conducted between the observed data and the respective equations.

The results obtained in this research, when analyzed with R^2^ and statistical indices, indicate that the Langmuir model provides a high fit for all experiments. These findings indicate that both the *E. crassipes* biomass and the bacterial cellulose biomass imply that these chemisorption reactions are characterized by a homogeneous and monolayer process, as previously documented in [25]. The Langmuir isotherm is the model that best represents these results, demonstrating that the maximum adsorption capacities will then be parameters used in a step-up process. These results confirm that these two biomasses could be used in heavy metal adsorption processes due to their suitability. Under the conditions studied, the interaction stage between the active sites of the adsorption biomasses and Cr (VI) reveals that this type of adsorbent has abundant adsorption sites (OH) that are easily accessible for the removal of different heavy metals present in water [58,59]. The adsorption processes and adjustments to the Langmuir isotherm indicate the involvement of chemisorption processes. Furthermore, the maximum adsorption capacities will serve as parameters in the context of a stepwise process, while the rate constant Kl will be employed in thermodynamic processes [60,61].

### 3.6. Kinetic Studies

**Figure 9:**
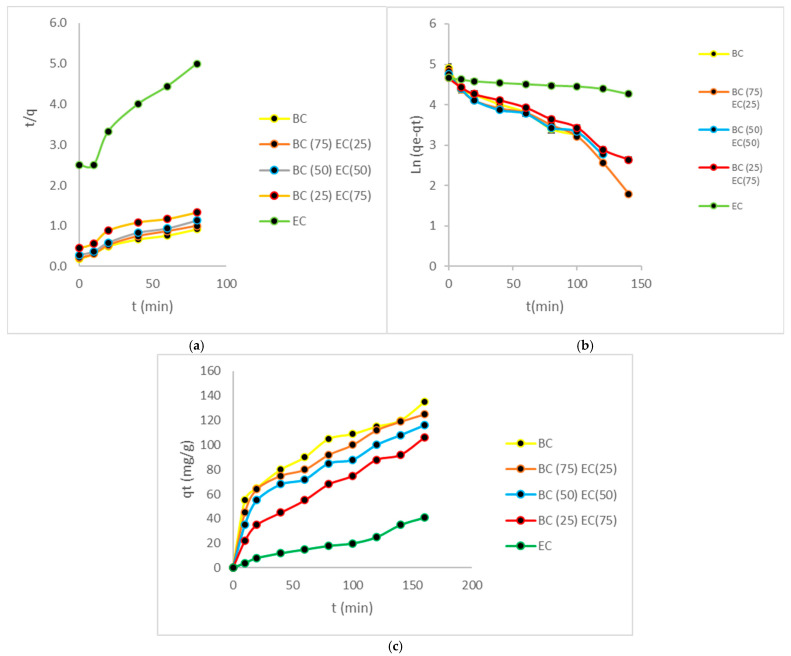
Various kinetic studies are represented by distinct graphs. (**a**) shows pseudo-first-order configuration, (**b**) shows pseudo-second-order configuration, and (**c**) shows capacities of adsorptions.

Kinetic studies are conducted in controlled environments; therefore, they were utilized in this study in batch, with the objective of predicting the adsorption rate. This was of crucial importance when modelling and designing this treatment system [16]. Previous studies demonstrated that these biomasses, especially those with large amounts of bacterial cellulose, have a high number of active sites, which generates a high adsorption rate in the first minutes. Figure 9 shows the various Kinetic studies represented by distinct graphs.

The experimental values for all biomasses in the Cr(VI) ion adsorption processes coincided with the values reported for the isotherms used previously, with these acting as two complementary models in the design processes of treatment systems. Through its representative biomasses, bacterial cellulose showed an adjustment to the second-order kinetics, with chemisorption being the most representative process in the adsorption of Cr(VI) metal ions, which involve valence forces or cation exchange between the active sites and this contaminant. BC biomass has an exchange rate of 1.22 × 10^−3^ g/(mg*min); the parameters are shown in Table 7.

The rate constant for BC(75)+EC(25) biomass was determined as 1.20 × 10⁻^3^ g/(mg*min). This result indicates the stability and adsorption capacity of the composite material formed by the interaction of bacterial cellulose and EC biomass. Furthermore, the BC(50)+EC(50) and BC(25)+EC(75) biomasses demonstrate interesting compatibility with the second-order model, accompanied by an adsorption rate of 1.20 × 10⁻^3^ g/(mg*min) and 1.16 × 10⁻^3^ g/(mg*min), respectively. In the context of the study, it was observed that biomasses exhibited a high degree of reactivity in the presence of Cr(VI), indicating the presence of abundant active adsorption sites that facilitate the removal of heavy metals. The second-order model was found to be a suitable fit, indicating the existence of an interaction between the biomass’s adsorption sites and Cr(VI), thereby controlling the process. This suggests the presence of a significant number of active sites on the surface of the adsorbent [33]. In contrast, plant biomass exhibited a better fit with the first-order model, which can be attributed to its comparatively lower adsorption capacity.

### 3.7. Process of Elutions

**Figure 10:**
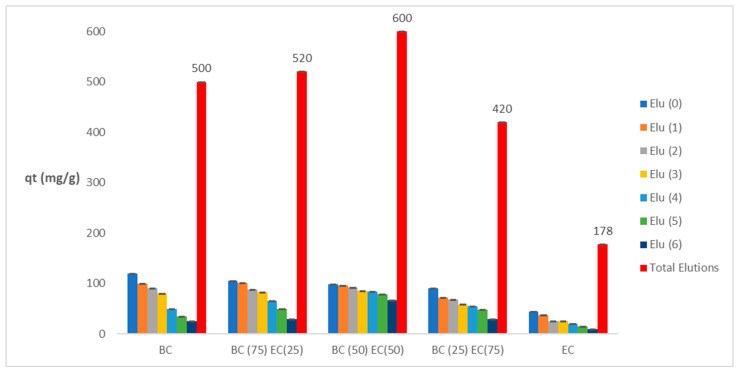
Illustration of various elution processes with EDTA, demonstrating total summation.

Desorption processes involving EDTA were conducted on the various biomasses. Figure 10 shows the various reuse instances for all biomass types, along with their respective elution and adsorption capacities. It should be noted that the final capacities depicted in this figure represent the aggregate sum of all individual capacities.

It is evident that bacterial cellulose biomass experiences a decrease in its adsorption capacity with seven cycles. Conversely, biomasses comprising a higher proportion of *E. crassipes* cellulose exhibit enhanced resistance to EDTA following each elution process. This enhanced resistance can be ascribed to the presence of lignin in the cellulose matrix, which contributes to resilience after elutions [62]. The biomass with the optimal capacity of 600 mg/g, designated as BC(50)+EC(50), is particularly well-suited for utilization in industrial wastewater treatment system assembly processes. The favourable regeneration and high adsorption capacity after elutions give *E. crassipes* biomass great applicability in heavy metal wastewater treatment [28,63]. The repeated use of *E. crassipes* biomass has been demonstrated in different adsorption–desorption cycles, offering significant advantages in terms of practical applications and profitability [64].

EDTA, a compound known to be effective in elutions of heavy metals, is able to bind with the metals through its hydroxyl groups, which become protonated in the process [65]. This reagent is considered to be one of the most effective due to its non-toxic nature and its applicability across various recycling cycles and subsequent removal processes of heavy metals [66]. It is highly effective in the chelation of heavy metals present in adsorbent biomasses, particularly cellulose [67]. The composition of Cr (VI) in the biomass and its elution with EDTA cause the wear of the bacterial cellulose biomass via a chelating agent [68]. Notwithstanding, the biomass remains available for subsequent adsorption cycles and further adsorption.

### 3.8. Treatment System Costs

In addition to achieving the desired effectiveness, the development of a treatment system must be economically viable at the time of its installation in contaminated bodies or in the treatment of effluents loaded with contaminants. For this reason, some characterizations of the materials used in this research were carried out to determine the economic and technical viability of this invention. Table 8 summarizes the three biomasses used in this research.

The cost of the drying, crushing, and logistics of obtaining *E. crassipes* is approximately 2 dollars per kilogram of biomass [1,25]. The cost of producing bacterial cellulose is around 25 dollars, taking into account all production implements [9,10]. EDTA costs 0.1 dollars when used in elutions [11].

The EC(50)+BC(50) biomass, exhibiting an adsorption capacity indicator of 42 g of Cr (VI) per dollar, is the most cost-effective due to the low cost of *E. crassipes* and the high capacity of bacterial cellulose. This combination of biomasses is optimal, given the resilience capacity of plant biomass and its low cost, coupled with the high capacity of bacterial cellulose. The second indicator is for the biomass EC(25)+BC(75), which has an adsorption capacity of 25 g/USD. This value is decisive when removing heavy metals at very low costs; however, if an ideal treatment system is required for high concentrations of heavy metals, the biomass BC(75)+EC(25) is recommended because the contaminant will be removed in less exposure times.

Table 9 shows some comparisons with the sum of adsorption capacities together with their production costs. The adsorption capacities, combined with the elutions and subsequent cost characterization of these adsorbent biomasses, result in an indicator of the quantity of contaminant in grams per dollar used to obtain this material. This indicator could be used to evaluate treatment systems with cellulose adsorbents. The following table illustrates a selection of typical examples of biomasses, both specialized varieties and those employed in isolation for the purpose of adsorption processes for certain heavy metals.

It is evident from data that the more specialized the adsorbent material, the greater its adsorption capacity and, consequently, the higher its direct production cost. Chitosan and *crassipes* biomasses emerge as the most economical options, though they exhibit comparatively lower adsorption capacity yields, with the indicator g/USD falling below 15 g/USD. Conversely, the most specialized cellulose and composite biomasses exhibit superior adsorption capacities but higher costs, demonstrating a coefficient ranging from 23 to 30 g/USD. It is notable that cellulose with polyaniline is the evaluation indicator that comes closest to operational viability, with an indicator of 30 g/USD. However, EC(50)+BC(50) biomass has the most suitable evaluation indicator, albeit within the context of Cr (VI) remediation processes.

## 4. Conclusions

In order to ascertain the most efficacious performance with regard to Cr (VI) adsorption, a series of experiments were conducted, the adsorption of which was evaluated using isotherms and adsorption kinetics. In addition, the ideal design criterion for adsorbent materials is the elution through EDTA, with a view to increasing the adsorption capacities and reducing the costs of these materials. It was determined that the material between bacterial cellulose and *E. crassipes* biomass should be shared equally. The rationale behind this choice is that bacterial cellulose possesses a cellulose content of 100%, while *E. crassipes* biomass demonstrates resilience to withstand more than eight treatment cycles. Moreover, the cost of this material is notably low. In instances where the treatment of industrial wastewater with a high flow rate and substantial Cr (VI) content is required, the utilization of EC(25)+BC(75) is recommended, owing to its proven efficacy in eliminating this contaminant. In scenarios involving less contaminated materials, yet high flow rates, the EC(50)+BC(50) system is proposed. Conversely, when a more cost-effective system is required and the water to be treated is less contaminated with a lower load, the EC(75)+ BC(25) system is considered. The utilization of these materials has been demonstrated to enhance industrial wastewater treatment processes, thereby contributing to Sustainable Development Goal 6 (SDG 6), which emphasizes the sustainability of water resources. These materials are particularly well-suited for the assembly of large-scale industrial wastewater treatment systems. Their development is straightforward, economical, and notably effective in the removal of heavy metals.

## Figures and Tables

**Table 1:** Models of isotherm and kinetics.

		Model Isotherm	
Capacity of adsorption	(1)	qe= $\frac{V(Ci-Cf)}{m}$	qe: (mg/g) adsorption capacity at equilibrium; V: volume (mL); m: biomass (g); Ci: initial concentration mg/L; Cf/L: final concentration.
Freundlich equation	(2)	$qe=K_{f}{\left(Cs\right)}^{n}$	qe: (mg/g) adsorption capacity; Cs: (mg/L) equilibrium concentration of adsorbents in solution; K_f_: (L/mg) constant for Freundlich.
Langmuir equation	(3)	qe= $\frac{qmsK_{l}Ce}{1+K_{l}Ce}$	qe: (mg/g) adsorption capacity at equilibrium; Ce: (mg/L) equilibrium concentration of adsorbents in solutio; qm: (mg/g) maximum adsorption capacity; K_l_: (mg/g) Langmuir constant.
		Model Kinetic	
Pseudo first order	(4)	$qt=qe\left[1-\exp\left(-K_{1}t\right)\right]$	qt and qe (mg/g) are uptake amount of pollutions at equilibrium and time t (h); K_1_(min^−1^) is adsorption rate constant of pseudo-first order.
Pseudo Second order	(5)	$qt=\frac{{qe}^{2}K_{2}}{K_{2}\left(qe\right)t+1}$	q_t_ and q_e_ (mg/g) are uptake amount of pollution at equilibrium and time t (h); K_2_ second-order model.

**Table 2:** Results of the analysis of the relationship between densities.

Biomass	Biomass m (g)	Volume (mL)	Density Biomass g/mL (ρ)	Particle (mg)	Volume Particle (mm)	Density Particle ρ(p)	$\varepsilon =1-\frac{p}{p(p)}$
BC	0.3	0.48	0.62	0.01	0.005	1.9	0.68
EC	0.3	0.35	0.85	0.01	0.005	2	0.57
EC(25)+BC(75)	0.3	0.46	0.65	0.01	0.005	2	0.67
EC(50)+BC(50)	0.3	0.40	0.75	0.01	0.005	1.9	0.62
EC(75)+BC(25)	0.3	0.38	0.78	0.01	0.005	2	0.60

**Table 3:** Composition of EC sample with Cr (VI).

Element	Weight	Percentages %
Carbon	49.2	48.7
Oxygen	38.2	37.2
Chromium	4.2	4.1

**Table 4:** Composition of BC sample with Cr (VI).

Element	Weight	Percentages %
Carbon	45.1	44.1
Oxygen	41.3	40.2
Chromium	10.0	9.1

**Table 5:** Summary of parameters.

Experiment 600 mg/L	EC(75)+BC(25)	EC(50)+BC(50)	EC(25)+BC(75)
Time of rupture (min)	120	160	200
Capacity of adsorptions (mg/g)	99 ± 6	116 ± 5	123 ± 6

**Table 6:** The isotherms are represented.

	Isotherm	Constante	R^2^
EC(25)+BC(75)	Langmuir	K_l_ = 1.1; q_ms_; 123	0.99
	Freundlich	K_f_ = 0.16	0.91
EC(50)+BC(50)	Langmuir	K_l_ = 0.9; q_ms_; 117	0.99
	Freundlich	K_f_ = 0.14	0.91
EC(75)+BC(25)	Langmuir	K_l_ = 0.7; q_ms_; 99	0.99
	Freundlich	K_f_ = 0.12	0.91

**Table 7:** Parameters of model kinetics.

	Pseudo First Order			Pseudo Second Order		
Samples	qt (mg/g)	K_1_ (min)	R^2^	qt (mg/g)	K_2_ g/(mg*min)	R^2^
BC	140	0.93	0.94	141	1.22	0.99
BC(75)+EC(25)	123	0.94	0.96	124	1.20	0.98
BC(50)+EC(50)	117	0.94	0.97	116	1.16	0.96
BC(25)+EC(75)	101	0.95	0.99	101	1.01	0.9
EC	45	0.99	0.99	46	0.75	0.9

**Table 8:** Cost of biomass g Cr/(USD).

Cost	BC	EC(25)+BC(75)	EC(50)+BC(50)	EC(75)+BC(25)	EC
Capacity of adsorptions (g Cr/kg material) Cost (USD) 1 kg material	500 ± 9 20	520 ± 11 18	600 ± 11 14	420 ± 10 12	178 ± 14 7
g Cr/(USD)	25	28	42	35	25

**Table 9:** Cost of biomass as per some references.

	Contaminant	Capacities of Adsorption mg/g	Cost (USD) 1 kg Material	g /(USD)	Reference
EC(50)+BC(50)	Cr (VI)	600	14	42	
Musk melon	Cu (II)	120	10	12	[69]
Banana peel	Cu (II)	25	5	5	[70]
Coconut shell carbon	Zn (II)	45	10	5	[71]
Alginate	Cr (VI)	238	25	10	[72]
Fly ash	Cu (II)	207	20	10	[73]
Chitosan beads	Cr (VI)	76	5	15	[74]
Bacterial cellulose/polyaniline	Cr (VI)	755	25	30	[75]
Poly(amidoxime)/bacterial	Pb (II)	1500	60	25	[76]
keratin from wastes to synthesize keratin/cellulose nanobiocomposite	Cd (II)	695	60	12	[77]
Clay_cellulose biocomposite	Pb (II)	389	30	13	[78]
BC/EDTA/ GO nano_composite membrane	Pb (II)	970	45	22	[79]
Magnetic chitosan composite (MCC)	Pb (II)	220	25	9	[80]

## Data Availability

The original contributions presented in this study are included in the article. Further inquiries can be directed to the corresponding author.
